# Endemic Jeffrey Pine Beetle Associates: Beetle/Mite Fungal Dissemination Strategies and Interactions That May Influence Beetle Population Levels

**DOI:** 10.3390/microorganisms9081641

**Published:** 2021-07-31

**Authors:** Javier E. Mercado, Beatriz Ortiz-Santana, Shannon L. Kay

**Affiliations:** 1 USDA/FS/Rocky Mountain Res. Sta., Fort Collins, CO 80526, USA; shannon.kay@usda.gov; 2 USDA/FS/Northern Res. Sta., Madison, WI 53726, USA; beatriz.ortiz-santana@usda.gov

**Keywords:** mutualist, antagonist, blue-stain fungus, fungal biome, population regulation

## Abstract

Fungal and mite associates may drive changes in bark beetle populations, and mechanisms constraining beetle irruptions may be hidden in endemic populations. We characterized common fungi of endemic-level Jeffrey pine beetle (JPB) in western USA and analyzed their dissemination by JPB (maxillae and fecal pellet) and fungivorous mites to identify if endogenous regulation drove the population. We hypothesized that: (1) as in near-endemic mountain pine beetle populations, JPB’s mutualistic fungus would either be less abundant in endemic than in non-endemic populations or that another fungus may be more prevalent; (2) JPB primarily transports its mutualistic fungus, while its fungivorous mites primarily transport another fungus, and (3) based on the prevalence of yeasts in bark beetle symbioses, that a mutualistic interaction with blue-stain fungi present in that system may exist. *Grosmannia clavigera* was the most frequent JPB symbiont; however, the new here reported antagonist, *Ophiostoma minus,* was second in frequency. As hypothesized, JPB mostly carried its mutualist fungus while another fungus (i.e., antagonistic) was mainly carried by mites, but no fungal transport was obligate. Furthermore, we found a novel mutualistic interaction between the yeast *Kuraishia molischiana* and *G. clavigera* which fostered a growth advantage at temperatures associated with beetle colonization.

## 1. Introduction

Phytopathogenic fungi, such as several blue-staining species associated with bark beetles, establish a variety of symbiotic interactions with beetles that range from obligate mutualisms to antagonisms. Most of our understanding of these interactions is derived from a few bark beetle species that undergo populations irruptions. A well-studied example in western North America is that of the blue-stain fungus *Grosmannia clavigera* (Rob.-Jeffr. & R.W. Davidson) Zipfel, Z.W. de Beer & M.J. Wingf. This fungus gains transport to suitable host trees aided by the mountain pine beetle (*Dendroctonus ponderosae*, Hopk.), hereafter MPB. In return, *G. clavigera* provides nutrition to the beetle’s larvae developing in the phloem, making their relationship mutually beneficial (mutualistic) to both organisms. In addition to nutrition, bark beetles derive other benefits from their fungal associates such as the detoxification of harmful oleoresin compounds [1]. Additionally, bark beetle chemical communication is partly mediated by yeasts, such as *Kuraishia capsulata* (Wick.) Y. Yamada, K. Maeda & Mikata and *Ogataea pini* (Holst) Y. Yamada, M. Matsuda, K. Maeda & Mikata, that synthesize and release verbenone used by searching beetles to recognize host saturation levels during beetle attack [2]. Given how fungi can influence their associated bark beetles, a good understanding of their prevalence during contrasting beetle population levels (i.e., collapsing and endemic vs. irruptive) can help elucidate their effect on shifting population levels; however, studies on collapsing or endemic-level bark beetle populations are rarely performed. Studies looking at symbiotic fungal communities of *Dendroctonus* bark beetles have found that their frequency can change over time with trends directly associated with fluctuations of their carrier beetle populations, strongly suggesting the type of interaction occurring. For example, an increase of *Ophiostoma minus* in the southern pine beetle (*Dendroctonus frontalis* Zimmermann) can drive a decline of the beetle’s population, representing an antagonistic interaction that regulates their population [3,4]. Conversely, in the MPB a decrease in the frequency of nutritional blue-stain fungi, which is abundant during epidemic-population levels, trended directly with a decline in the insect’s population size, signaling a mutualistic interaction between the two organisms [5]. Therefore, monitoring the trends of important fungal associates in irruptive bark beetles during population shifts could help explain and perhaps predict fluctuations of bark beetle populations.

The ways in which blue-stain fungi disseminate their spores between carrying beetles and host trees are varied, and identifying if evolutionary adaptations of insects, phoretic mites, or the fungi influence their frequency in these systems could aid in determining the type of symbiotic interaction being established. Fungi associated with *Dendroctonus* bark beetles can be carried internally or externally on beetles, or by their phoretic mites [6,7]. Yeasts can be carried in the beetle’s digestive tract, especially in the gut [8,9], yet other forms of dissemination may exist. For instance, we know that fungi can survive enzymatic action in the gut of MPB [10], and although their survival of digestive elimination has been suggested [11], this is not fully understood.

One closely related species to the MPB is the Jeffrey pine beetle (*Dendroctonus jeffreyi*, Hopk.), hereafter JPB. The two species resemble each other, both morphologically and genetically [12,13,14,15]. These two species also share a unique trait within the genus *Dendroctonus*, the possession of a pit or mycangium (specialized fungus harboring site) contained within each maxilla (maxillae in text) for the dissemination of fungi [16]. Besides the above morphological similarities, these two bark beetles also rely on the common nutritionally beneficial fungus *G. clavigera*, which complements the nutrients they obtain from the phloem. Although MPB also obtains nutritional benefits from *Leptographium longiclavatum* S.W. Lee, J.J. Kim & C. Breuil and *Ophiostoma montium* (Rumbold) von Arx, its phoretic mites also carry them, making its mutualism with *G. clavigera* one facultative. *Grosmannia clavigera* is the only known nutritional blue-stain fungus of JPB and we do not know if its phoretic mites carry it. This makes the type of mutualism between JPB and *G. clavigera* one obligate. Additionally, both species can carry a variety of other fungi and yeasts that have unresolved effects on their populations.

As in several other bark beetle species, JPB fungal associates have been studied during moderate population irruptions, when access to adequate samples is best. Nine fungi are associated with JPB (Table 1), of which the blue-staining *G. clavigera* is commonly carried by the beetle across most of its range [17]. But the other two MPB blue-stain fungal mutualist, *O. montium* and *L. longiclavatum,* were reported each once before from JPB in California [18,19]. The long, clavate, and septate conidia morphologically distinguish them from most related taxa, but separating them often requires molecular identification. Therefore, as in the MPB, *L. longiclavatum* may be more prevalent in JPB populations than expected [5]. Most yeasts transported by JPB belong to the Order Saccharomycetales, of which *K. capsulata* and *Nakazawaea holstii* (Wick.) Y. Yamada, K. Maeda & Mikata are the most prevalent [20]. Although the function of many yeasts in bark beetle symbiotic systems is not completely understood, their ubiquity suggests that many of these are at least commensals, benefiting by gaining transport to new hosts with the beetles. In culture and trees, yeasts often grow together with blue-stain fungi which makes separating them during isolations difficult [21], but their tolerance of each other suggests that a non-antagonistic symbiotic relationship may exist between them.

In this study, we examined the composition and frequency of associated fungi in an endemic JPB population sample. We isolated fungi and yeasts from JPB maxillae, rectal fecal pellets, and mites. We aimed to identify relationships between the frequency of associated fungi to the beetle’s endemic-population level, using their dissemination method and known function in other bark beetle systems to derive a better understanding of their role in the JPB system. Based on previous work and the endemic-population level of our sample, we hypothesized that: (1) as a population regulation factor noted in the MPB, *G. clavigera* would either be less abundant than in non-endemic populations or that another fungus may be more prevalent; (2) as in other *Dendroctonus* species, JPB would transport primarily mutualistic fungi, while the associated fungivorous mites would transport primarily non-mutualistic fungi to JPB, and (3) based on the prevalence of yeasts in bark beetle symbioses, that a mutualistic interaction with blue-stain fungi commonly present in the system may exist. Our findings would not only increase our knowledge about JPB associates but would reinforce the hypothesis suggesting that an endogenous population control of irruptive bark beetle populations is exerted by their microbial associates.

## 2. Materials and Methods

### 2.1. Bark Beetle Sample Collection

**Figure 1:**
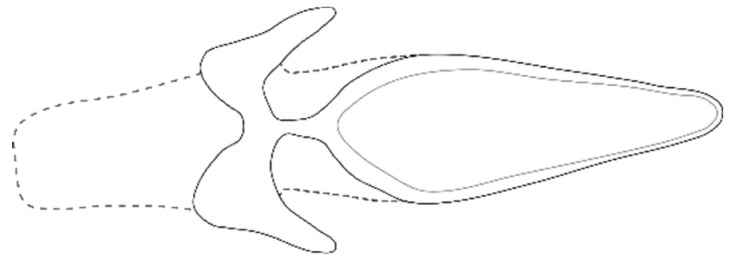
We used the undescribed endophallus of Jeffrey pine beetle (illustrated) as new character to distinguish this species from the similar mountain pine beetle and found it to be a useful character.

Jeffrey pine beetles were collected in the Lassen N.F. on 23 June 2017. Jeffrey pine beetle populations fluctuate between endemic and moderately irruptive levels. In the general area around the collection site, recent JPB populations were moderately irruptive from 1991 to 1997, 2001 to 2004, and 2014 to 2016. At the time of collection, the species activity was experiencing a declining trend relative to their climax in 2016 [22]. Specific to our sample, the declining population was considered to be endemic, given the difficulty in finding trees affected by the beetle and its attack behavior. Fifty-seven beetles were individually collected from beneath the bark of a 300-year-old Jeffrey pine before their early summer dispersal flight period. Beetles were immediately stored in a cooler and shipped refrigerated to the Rocky Mountain Research Station in Fort Collins. Due to their similarity with the MPB and their overlapping range, we carefully identified JPB beetles using available morphological identification keys [12]. We compared the genitalia (male endophallus) to that of *D. ponderosae* and found it to be different. We illustrate JPB’s endophallus here for the first time (Figure 1).

### 2.2. Isolation of Fungi from Beetle Structures and Mites

We thawed beetles for four minutes before dissections to remove their maxillary cardines, fecal pellets, and fungivorous mites, if present. We cultured the pellets and mites directly into the agar plates without performing a rinse, whereas two sequential rinses on a drop of molecular grade sterilized water were performed on exterior surfaces of the maxillary cardines (maxillae hereafter) before culturing. We used malt extract agar (1.5% MEA, Bacto Malt Extract, BD Biosciences, San Jose, CA, USA) for the direct culture of the three targets to obtain prevailing JPB fungal associates. We used a direct culture method instead of sequential dilutions and streaking method since competition between common fungal associates of related *Dendroctonus* beetles is non-significant [23]. We examined cultures daily for 21 days to recover any distinct fungal colonies.

### 2.3. Single Fungal Strain Isolations

In *Dendroctonus* bark beetle’s fungal isolates, yeasts are typically the first associates to grow. These usually surround other fungal growth making their separation difficult. We tried two methods to recover fungi from yeast overgrowth: single spore isolation and hyphal tip harvesting, of which the latter was used for this study. Material for single spore isolation came from two sources, direct spore transferences from aerial conidiophores growing on MEA and sterilized phloem sections (2 × 4 cm) placed inside sterile and parafilm sealed 60 mm petri dishes. We harvested hyphal tips from above as well as from below the agar. This last strategy was the most efficient in reducing yeast contamination when harvesting hyphal tips.

### 2.4. Morphological Determination

We studied cultural and microscopic characters of pure strains and made identifications based on authoritative sources [24,25]. Strains’ cultural and morphological characters used in diagnostics were color, growth characteristics of the margin, and patterns of spore production in MEA plates. In addition, from conidia produced in the sterilized phloem sections, the microscopic diagnostic characters used were conidiophore type and length, conidial shape, and ratio. We identified fungivorous mites only to the Family taxonomical level. All isolates were deposited in the Center for Forest Mycology Research Culture Collection (CFMR) in Madison, WI, USA.

### 2.5. Molecular Data

In the present study, we generated DNA sequences of the nuclear ribosomal ITS2-LSU (partial 5.8 + ITS2 + partial 28S) and the protein-coding gene β-tubulin (partial gene) from 21 cultures. We performed DNA extraction, amplification, and sequencing as indicated in Mercado and others [5]. DNA sequences were used for molecular identification and phylogenetic analyses. A total of 20 ITS2-LSU and β-tubulin sequences were newly generated while 35 ITS2-LSU and 44 β-tubulin were retrieved from GenBank. The selection of sequences from GenBank [26] was performed using BLASTn search results and species previously grouped within the *Grosmannia* and *Ophiostoma* clades [27,28,29,30,31]. The information for these isolates is provided in Table 2. Newly generated sequences were edited with Sequencher 4.8 (Gene Codes Corp., Ann Arbor, MI, USA) and were deposited in GenBank (MZ297376-MZ297395, MZ296739-MZ296755).

### 2.6. Sequences Alignment and Phylogenetic Analyses

To align the DNA sequences and to construct our phylogenetic trees we proceeded as follows. Bayesian inference (BI) and Maximum likelihood (ML) phylogenetic analyses were executed on two datasets: *Ophiostoma* ITS2-LSU + β-tubulin and *Grosmannia* ITS2-LSU + β-tubulin. DNA sequences were aligned using Clustal W2.1 through CIPRES Science Gateway [32] and were manually adjusted with AliView 1.18 [33]. Final alignments were deposited in TreeBASE (TB2:S28351, S28352). The loci were concatenated using Sea View 4 [34] and the two-locus datasets were appended with a MrBayes block, partitioned the data by gene, codon position, and noncoding region for BI analysis. The nexus files were converted into phylip files and run through jModelTest 2 [35] in CIPRES to estimate the best substitution model for the analyses. The best-fit model estimated for the *Ophiostoma* ITS2-LSU + Btubulin dataset was TrN + G (corresponding to SYM in MrBayes with parameters nst = 6 and rates = gamma), whereas for *Grosmannia* ITS2-LSU + Btubulin dataset was TrN (corresponding to SYM in MrBayes with parameters nst = 6 and rates = equal). Bayesian inference analysis was performed using MrBayes 3.2.2 [36] on XSEDE through CIPRES. The parameters mentioned above were used for each dataset for 1,000,000 generations in two runs and four chains with trees sampled every 100 generations. The burn-in period was set to 0.25. Maximum likelihood analyses were performed using RAxML-HPC2 on XSEDE through CIPRES under the GTR model with gamma distributed rate heterogeneity and 1000 rapid bootstrap replicates; other parameters were kept at their default settings. Phylogenetic trees were visualized and edited in FigTree 1.4.4 [37] and rooted to midpoint. Final trees were edited in Adobe Illustrator CC 2018 (San José, CA, USA). Strong support values of clades are >90% in ML and >0.95 posterior probabilities (PPs) in BI analyses, whereas moderate support values are >70% and >0.90, respectively. The bootstrap frequencies (>50%) and posterior probabilities (>0.80) are shown on branches.

### 2.7. Prevalence of Fungi in Beetle Maxillae and Pellet, and in Mites

We analyzed the prevalence of two fungal species in two different locations on the beetle, by fitting a generalized linear mixed model (GLMM) with a binomial response and logit link using the fixed effects of fungal species and location, as well as their interaction. Since each beetle had four observations, we included a random beetle effect in the model. Similarly, we fitted a GLMM model to the mite data using species as a fixed effect and beetle ID as a random effect. We performed these analyses in R version 4.0.5 [38].

### 2.8. Fungal Growth Rates across a Temperature Gradient and Interactions with Yeast

We grew eight (*O. minus*) and nine (*G. clavigera*) pure strain subsamples of the characterized blue-stain fungi in incubators (Thermo Scientific™ Precision 818, Marietta, OH, USA) at 5 °C intervals from −10 to 30 °C and at 2 °C intervals from 32 to 36 °C. We cross-marked the MEA plates at 90 °C and averaged two perpendicular diameter measurements to estimate the growth of every single strain of blue-stain fungi and of a replicate that had been allowed to grow for 7-days with the most prevalent yeast species. Physiological characters measured included the temperature at which minimum, maximum, and optimal growth rates were achieved by each blue-stain fungus, and in combination with the yeast. We used a Gaussian GLMM with a random effect for strain to fit the growth measurements to the predictors of fungal species, categorical temperature increment, whether yeast was present, and an interaction between all three variables.

## 3. Results

### 3.1. Phylogenetic Analyses

**Figure 2:**
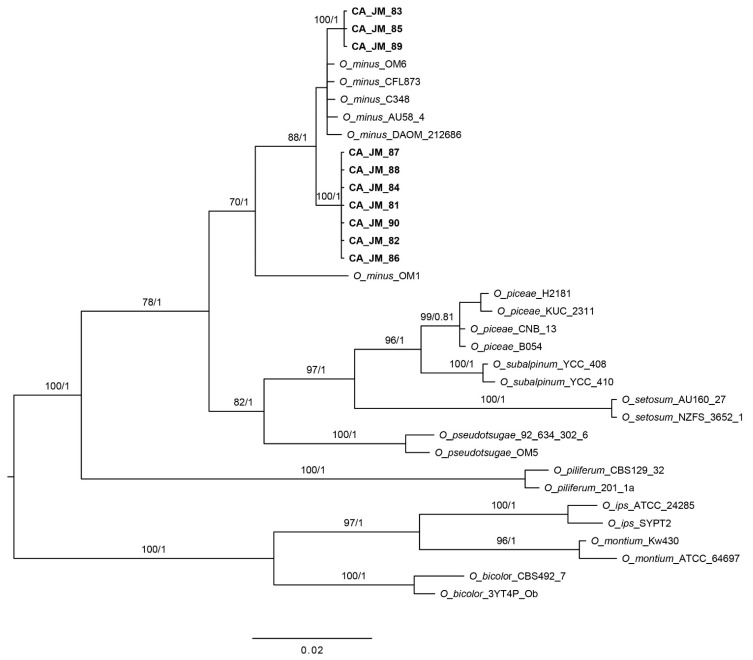
Phylogeny of *Ophiostoma* species as inferred from BI analyses of ITS2-LSU and B-tubulin sequences. Support values along branches are from ML Bootstrap (≥50%) and Bayesian posterior probabilities (PP ≥ 0.80), respectively.

**Figure 3:**
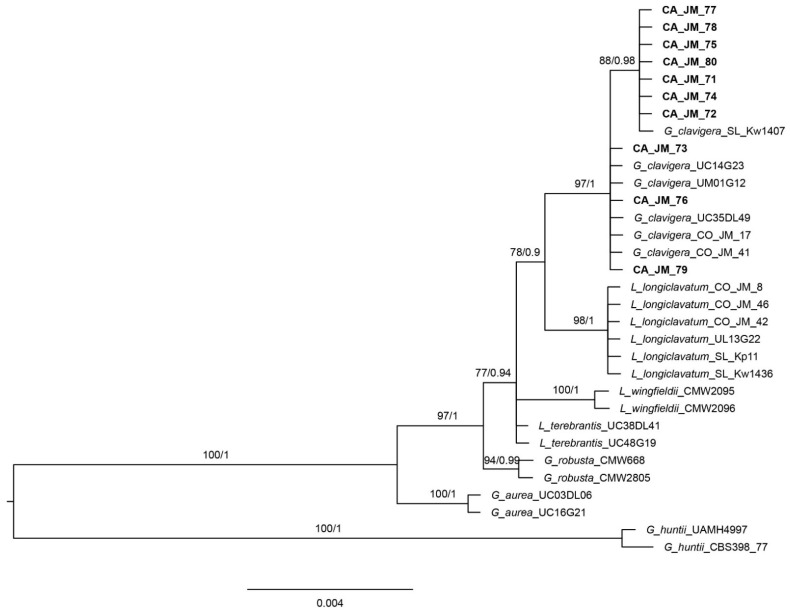
Phylogeny of the *Grosmannia* clade as inferred from BI analyses of ITS2-LSU and B-tubulin sequences. Support values along branches are from ML Bootstrap (≥50%) and Bayesian posterior probabilities (PP ≥ 0.80), respectively.

The combined *Ophiostoma* ITS2-LSU + Btubulin dataset consisted of 34 ingroup sequences with a total of 1566 positions (961 for ITS2-LSU and 605 for β-tubulin) while the *Grosmannia* ITS2-LSU + Btubulin dataset consisted of 32 ingroup sequences with a total of 1380 positions (871 for ITS2-LSU and 509 for β-tubulin). In the analyses of the *Ophiostoma* dataset, JPB isolates clustered in two clades: one consisting of three JPB isolates and *O. minus* isolates from Canada and the United States, and another consisted only of JPB isolates (Figure 2). When we compared β-tubulin sequences within the isolates of the first clade, we found that they differed in 4–6 nucleotides, whereas when we compared isolates between the two clades, they differed in about 17 nucleotides. Thus, isolates from the first clade are closely related to *O. minus*, while the other clade may not represent *O. minus*. In addition, isolate OM1 from the UK differed from isolate OM6 in more than 60 nucleotides. This suggested that this isolate may not have represented *O. minus*. When we compared the ITS2-LSU sequences, we found only three differences between these two clades. However, we were able to compare only portions of the ITS2 region since there were not complete ITS2-LSU sequences for *O. minus* available in GenBank. In the analyses of the *Grosmannia* dataset (Figure 3), all the isolates obtained from JPB clustered within the *Grosmannia clavigera* clade. The *Grosmannia clavigera* clade consisted of two groups, but these isolates differed only in one nucleotide in the ITS2-LSU dataset with no differences in the β-tubulin dataset. This confirmed that these JPB isolates likely represent *G. clavigera*.

**Figure 4:**
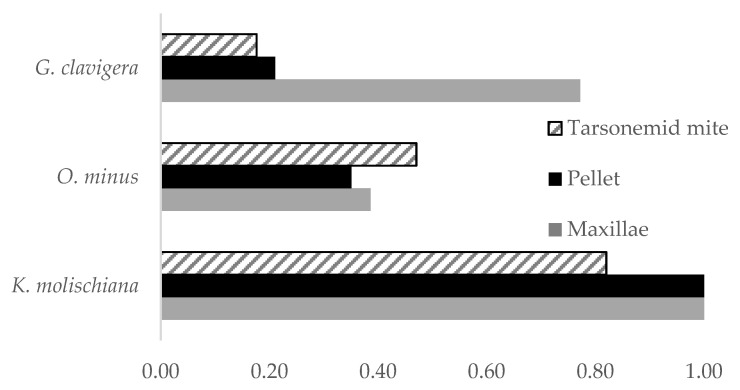
Percent frequency of three Jeffrey pine beetle fungal associates in tarsonemid mites, pellet, maxillae, from an endemic-population sample.

The most prevalent microorganism we recovered from JPBs maxillae, pellet, and mites was a yeast (Figure 4). Based on cultural characters of the yeast we separated from uncommon species present in the sample. From these, we sequenced strain CA-JM-92 obtaining only the ITS sequence which was compared with other species using BLAST Search tool (the nearest match for this isolate was the strain CBS:9990, KY103918, with a 99% Percent Identity and Query Cover of 97%). Using molecular data, we identified the yeast as *Kuraishia molischiana* Dlauchy, G. Péter, Tornai-Leh. & Kurtzman [39]. This species was recently split from *K. capsulata*, which was the only *Kuraishia* spp. previously reported from JPB. We also recovered two blue-stain fungi frequently, these represented an *Ophiostoma* and a *Leptographium* (a common anamorph of *Grosmannia*) species. We did not find any other previously documented JPB blue-stain fungal associates. Morphological and molecular identification (Figure 2) indicated that the *Ophiostoma* species represented *O. minus*. However, the *Leptographium* did not agree morphologically with all characters of *G. clavigera* as none of the strains produced the long, clavate, and septate conidia characteristic of *G. clavigera*, but molecularly matched that species closely (Figure 3). Although we found a high frequency of *G. clavigera* in our sample, we found the antagonistic *O. minus* in moderate frequency. The latter species is an indicator of collapsing southern pine beetle populations in southeastern USA [4,40].

### 3.2. Prevalence of Fungal Associates on Beetles and Mites

*Grosmannia clavigera* was most prevalent in the maxillae (probability of presence = 0.79), and less prevalent in the pellet (probability of presence = 0.34) and this difference was significant (Table 3). However, *O. minus* had a similar probability of presence in each location (Table 3).

**Figure 5:**
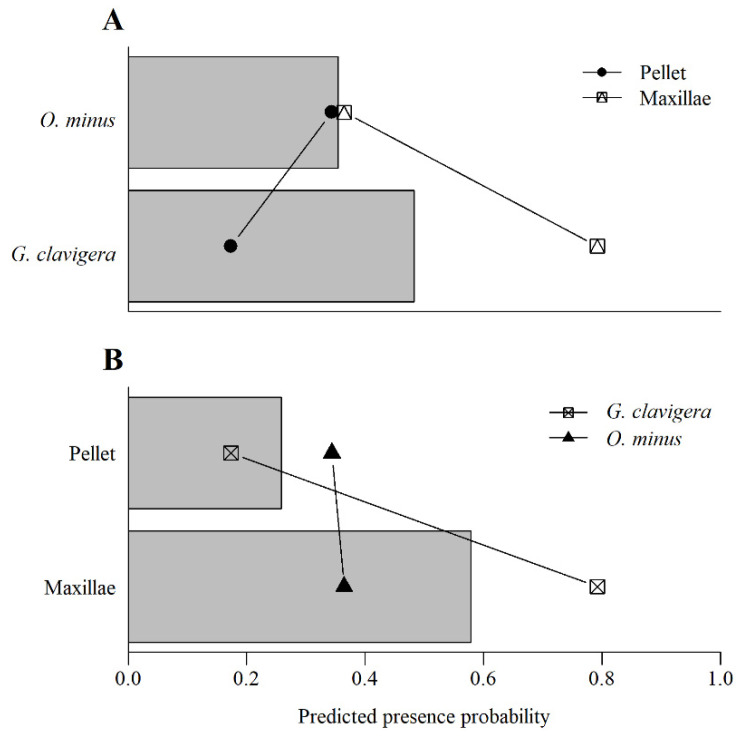
Predicted probability of presence of fungal associates *O. minus* and *G. clavigera* with the location interaction (**A**) and predicted probability of presence within by location with the fungal associate interaction (**B**). Main effects (averaged across species or location) are shown by bars while interaction effects, which allowed each fungal species to differ in presence between locations, are shown by points and lines.

The presence of both fungi, as well as the yeast in fecal pellets, indicated their capacity of surviving digestion; however, their prevalence in these samples varied. The yeast *K. molischiana* was present in both beetle structures and only slightly less on mites which suggested its capacity to exploit many dispersal strategies. Similarly, *O. minus* rate of recovery from the pellet was not different from its occurrence on the JPB maxillae (Table 3, Figure 5). In contrast, *G. clavigera* was significantly less likely to show up in the fecal pellets than in the maxillae (Table 3). Overall, there was a low frequency of blue-stain fungi on mites (Figure 4). However, we found mites to carry *O. minus* more frequently than *G. clavigera* (χ^2^ = 7.07, *p* ≤ 0.01, *n* = 17), and overall, within the system *O. minus* was more abundant on mites.

### 3.3. Yeast Interaction with Blue-Stain Fungal Growth across a Temperature Gradient

**Figure 6:**
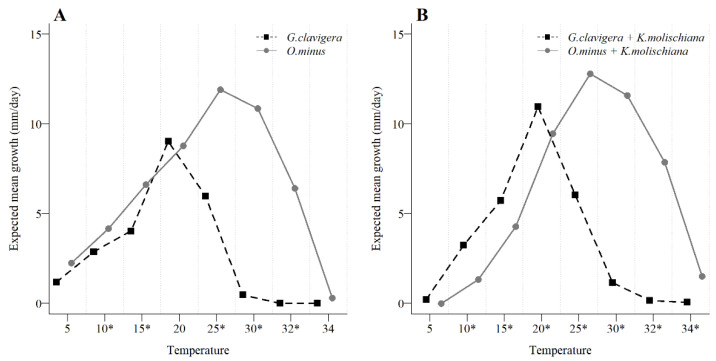
The expected average growth of nine isolates of the fungi *Grosmannia clavigera* and eight of *Ophiostoma minus* (**A**) and after their paired growth with the yeast *Kuraishia molischiana* (**B**), showed that while growing alone *O. minus* generally had a growth advantage over *G. clavigera* that reversed from 5 to 15 °C when both fungi grew in combination with the yeast. Significant differences are indicated with an asterisk (*).

In general, *O. minus* grew well over a wider range of temperatures than *G. clavigera*, especially in temperatures above 20 °C. In fact, the optimal growth temperature of *G. clavigera* was lower at 19.24 °C than the 25.06 °C of *O. minus* and that did not change when growing in association with the yeast *K. molischiana*, when these were 19.05 °C and 24.90 °C, respectively. We found that when not paired with the yeast, the growth of the two blue-stain fungi was nearest from 5 to 20 °C (see significant differences in Figure 6A), but *O. minus* maintained a growth advantage from 5 to 15 °C (Figure 6A). However, an inverse growth advantage, benefiting *G. clavigera* over *O. minus*, was evident in that same temperature range (not significant at 5 °C) when each blue-stain grew in association with the yeast (Figure 6B). However, above 20 °C, there was a small advantage in the growth of *O. minus* contrasting to when it grew alone (Figure 6B).

## 4. Discussion

Examining symbiotic associations of bark beetles from populations that are in equilibrium (endemic-level) or decreasing, often requires considerably more effort than studying irruptive ones, but only by studying endemic populations can we better understand the factors constraining them. Among *Dendroctonus* bark beetles, the Jeffrey pine beetle is the closest morphologically, genetically, and ecologically to the MPB, thus factors affecting its ecology may be similar to those in the more ecologically impactful MPB, making our findings relevant to understanding the MPB. For the first time, here we describe the fungal associates of an endemic JPB population. Although small in comparison, our study results contrast with a previous study that examined the fungal fauna of JPB across its distribution from a non-endemic population [17] by detecting the presence of an antagonistic fungus associated with its symbiotic system. The population level in the previous study was moderately high based on reports [22], as well as from personal comments made by Diana Six (17 February 2018), in contrast to our studied population.

As also found by Six and Paine [17], we found *G. clavigera* to be the most prevalent blue-stain fungal associate carried in the beetles’ maxillae in our endemic population sample. This is important since in the MPB symbiosis, *G. clavigera* is required (obligate mutualist) by beetles to reach their adult phase [41] and its function in the JPB symbiotic system is either similar or even more important, given the phylogenetic proximity of both insects and their fungi, and especially since this is the only mutualistic blue-stain fungus in that symbiosis. It is also important since a reduction of this and other MPB mutualists might be associated with a reduction in population size leading to endemic population levels [5].

Although showing molecular affinity to *G. clavigera*, strains obtained from JPB differed morphologically in that their conidia were never typical for the species, but similar observations were reported previously [17]. To verify if the growth media was affecting our observations, we cultured *G. clavigera* from MPB voucher isolates in our MEA media and contrary to our observation from JPB isolates obtained vigorous production of its typical conidia. We saw the same lack of typical conidia production when we cultured *G. clavigera* in ponderosa pine phloem; however, this has a different oleoresin composition from Jeffrey pine that could have affected our observations. Moreover, Paine and Hanlon [42] found that α-pinene, a primary component in the oleoresin of ponderosa pine reduced the growth of *G. clavigera* isolates from JPB while *n*-heptane, the principal oleoresin component of Jeffrey pine enhanced its growth. Therefore, it is probable that, as in their beetle carriers, *G. clavigera* in these two bark beetles may be differentiating into distinct species and further studies contrasting specimens from the two beetles may show that to be the case.

Finding *O. minus* was surprising. This fungus is antagonistic to the southern pine beetle, causing their populations to collapse when the beetle’s phoretic tarsonemid mites transporting the fungus increase in number [4]. This new record of blue-stain fungi from JPB may have resulted from the lateral transmission from mites carried to the attacked tree by *Ips emarginatus* LeConte, as tarsonemid mites and *O. minus* are carried by that species [18]. This bark beetle was present in the tree from which our collection was made but not sampled in our study (Danny Cluck, personal comment). Tarsonemid mites in our sample were scarce and many did not carry fungi, but mites carried *O. minus* more often than *G. clavigera* (Figure 4). Therefore, it is probable that the recruitment of *O. minus* by JPB is facilitated by phoretic mites moving from *I. emarginatus* to the beetle. Moreover, once within the phloem, *O. minus* is ingested by JPB and can become dispersed within the beetle’s niche making it more prevalent, reducing the reproductive success of the beetle. Confirming the lateral transmission of *O. minus* carried *by I. emarginatus* phoretic tarsonemid mites into the niche of the JPB could have important management implications. For example, we could attempt to reduce the reproductive success of JPB by influencing *I. emarginatus* to attack trees under the attack of JPB. Additionally, the presence of *I. emarginatus* in trees attacked by JPB may help us predict future shifts in JPB populations. A question that emerges from these findings is, what mechanisms influence the prevalence or reduction of *O. minus* from JPB populations? Future research should include analyzing fungal associates in different JPB population levels as well as include the sampling of *I. emarginatus* fungal associates and should relate findings to weather factors such as temperature and the ecosystems relative humidity which drive variations in fungal diversity. This will strengthen the hypothesis that antagonistic fungi act as endogenous bark beetle population regulators and what may drive their prevalence.

The topic of bark beetle fungal associates’ survival from digestion remains largely unknown, especially when looking at it as a means for their dissemination. Rivera and others [9] described the occurrence of yeasts in specific gut areas in several *Dendroctonus* species. They were able to grow species found in other *Dendroctonus* species hindguts, but their efforts were not aimed at detecting yeast dissemination strategies. The ubiquity of the yeast *K. molischiana* in both mycangia and pellet, as well as in the mites, suggests that this yeast does not have a dependency on beetles or mites for its dispersion and that it can exploit elimination. Dissemination of blue-stain fungi is also poorly understood. Dissemination of *O. minus* by JPB is secondary to its mite dissemination and its high survival rate in pellets, inferring that ingestion is similar to its prevalence in the mycangia suggest dissemination in beetle’s elimination is exploited by this fungus. Its unspecialized dissemination strategy suggests that as *K. molischiana*, it has a lack of dependence on JPB for its survival. In contrast to these two species, the higher frequency of *G. clavigera* in maxillary mycangia combined with its higher reduction during beetle elimination reflects the strong association of this species to JPB. These findings suggest that understanding more about the dissemination strategies of bark beetle associates may be useful to understand their role in their carrier beetle’s symbiosis but that complex interactions require further experimentation to be discerned. As hypothesized, a mutualistic fungus *G. clavigera* was more prevalent in JPB especially in what is considered a highly derived structure for mutualists dissemination, the mycangia, while a different fungus, in this case, a known *Dendroctonus* antagonist was more prevalent in mites.

Given the limitations of our study, the way in which *K. molischiana* affects the performance of *G. clavigera* deserves to be investigated in further detail. Yeasts such as *K. molischiana* cover the growth of filamentous fungi completely in culture, making their isolation difficult. Although we did not study the insulating effects of yeast coating in fungi at cold temperatures, it is possible that its compounds may provide such protection to *G. clavigera*. However, this does not entirely explain why a similar effect is not seen in *O. minus.* Thus, the thermal requirements of this warm temperature-loving species may not be met by the potential insulation provided by the yeast. However, this does not mean that the interaction of *O. minus* with the yeast is entirely antagonistic and further studies should focus on defining their relationship. Nevertheless, a mutualistic interaction was evident between *G. clavigera* and *K. molischiana* that may improve tree host establishment in priority of other fungal species, in this case, *O. minus*. The interaction is also indirectly mutualistic with JPB as it improves the incidence of its mutualist fungi over an antagonist. Our findings set a start point for further studies that will continue elucidating the complexity of these and similar interactions between insect microbial associates.

## Figures and Tables

**Table 1:** Previously documented fungal associates of Jeffrey pine beetle and the structure from which they have been collected.

Species	BeetleStructure	Locality	Reference
*Ophiostoma ips*/*montium*	*unreported*	Stanislaus NF, CA	[18]
*Grosmannia clavigera*	Maxillae	S. Bernandino Mnts., CA and NV	[17]
*Leptographium longiclavatum*	Maxillae	Monitor Pass, Toiyabe NF, CA	[19]
*Candida silvicola*	Gut	Northern California	[20]
*Diutina rugosa*	Gut	Northern California	[20]
*Kuraishia capsulata*	Gut	Northern California	[20]
*Nakazawaea holstii*	Gut	Northern California	[20]
*Priceomyces haplophilus*	Gut	Northern California	[20]
*Spencerozyma crocea*	Gut	Northern California	[20]

**Table 2:** Taxa, isolate information, location, and GenBank accession numbers of the samples used in the phylogenetic analyses. New sequences appear in bold.

Species	Isolate	Location	ITS-LSU	β-tubulin
*Grosmannia aurea*	UC03DL06	Canada-AB	GU370267	GU370181
	UC16G21	Canada-AB	GU370293	GU370207
*G. clavigera*	CA-JM-71	USA-CA	**MZ297376**	**MZ296739**
	CA-JM-72	USA-CA	**MZ297377**	**MZ296740**
	CA-JM-73	USA-CA	**MZ297378**	**MZ296741**
	CA-JM-74	USA-CA	**MZ297379**	**MZ296742**
	CA-JM-75	USA-CA	**MZ297380**	**MZ296743**
	CA-JM-76	USA-CA	**MZ297381**	**MZ296744**
	CA-JM-77	USA-CA	**MZ297382**	**MZ296745**
	CA-JM-78	USA-CA	**MZ297383**	**MZ296746**
	CA-JM-79	USA-CA	**MZ297384**	**MZ296747**
	CA-JM-80	USA-CA	**MZ297385**	**MZ296748**
	CO-JM-17	USA-CO	KY940832	KY940842
	CO-JM-41	USA-CO	KY940833	KY940843
	SL-Kw1407	Canada-BC	AY544615	AY263195
	UC14G23	Canada-AB	GU370266	GU370180
	UC35DL49	Canada-AB	GU370288	GU370202
	UM01G12	Canada-AB	GU370274	GU370188
*G. huntii*	CBS398.77	Canada	AY707208	AY707194
	UAMH4997	Canada	AY544617	AY349023
*G. robusta*	CMW 668	USA-ID	AY553397	AY534945
	CMW 2805	USA-ID	AY553396	AY534944
*Leptographium longiclavatum*	CO-JM-8	USA-CO	KY940824	KY940834
	CO-JM-42	USA-CO	KY940826	KY940836
	CO-JM-46	USA-CO	KY940830	KY940840
	SL-Kp11	Canada-BC	AY816687	AY816712
	SL-Kw1436	Canada-BC	AY816686	AY288934
	UL13G22	Canada-BC	GU370300	GU370214
*L. terebrantis*	UC38DL41	Canada-AB	GU370295	GU370209
	UC48G19	Canada-AB	GU370272	GU370186
*L. wingfieldii*	CMW 2095	France	AY553400	AY534948
	CMW 2096	France	AY553398	AY534946
*Ophiostoma bicolor*	3YT4P-Ob	Canada	DQ268606	DQ268637
	CBS492.7	USA-CO	DQ268604	DQ268635
*O. ips*	ATCC-24285	Canada	AY194937	AY194949
	SYPT2	Canada	AY194940	AY194955
*O. minus*	AU58.4	Canada-BC	—	AY548743
	C348	Canada	AY542496	AY542507
	CFL873	Canada	KC305144	KC336019
	DAOM-212686	Canada	—	AY305690
	OM1	UK	AY542494	AY542505
	OM6	USA-NE	AY542498	AY542509
	CA-JM-81	USA-CA	**MZ297386**	**MZ296749**
	CA-JM-82	USA-CA	**MZ297387**	**MZ296750**
	CA-JM-83	USA-CA	**MZ297388**	**MZ298931**
	CA-JM-84	USA-CA	**MZ297389**	**MZ296751**
	CA-JM-85	USA-CA	**MZ297390**	**MZ298932**
	CA-JM-86	USA-CA	**MZ297391**	**MZ296752**
	CA-JM-87	USA-CA	**MZ297392**	**MZ296753**
	CA-JM-88	USA-CA	**MZ297393**	**MZ296754**
	CA-JM-89	USA-CA	**MZ297394**	**MZ298933**
	CA-JM-90	USA-CA	**MZ297395**	**MZ296755**
*O. montium*	ATCC-64697	Canada	AY194941	AY194957
	Kw430	Canada	AY194948	AY194964
*O. piceae*	B054	Austria	HQ115733	—
	CNB-13	Spain	AJ538341	—
	H2181	Japan	AF211844	AB934362
	KUC-2311	Korea	—	DQ868379
*O. piliferum*	201/1a	United Kingdom	—	AF221629
	CBS129.32	Netherlands	—	AY305705
*O. pseudotsugae*	92-634/302/6	Canada-BC	AY542502	AY542510
	OM5	USA-ID	AY542500	AY548744
*O. setosum*	AU160-27	Canada	—	AY789160
	NZFS-3652-1	New Zealand	—	AY789159
*O. subalpinum*	YCC-408	Japan	—	AB200429
	YCC-410	Japan	—	AB200430

**Table 3:** *Grosmannia clavigera* was significantly more likely to be present in Jeffrey pine beetle’s maxillae than in its fecal pellet, while *Ophiostoma minus* was equally likely to be found on either.

Fungal Species	Value	DF	Chisq	Pr (>Chisq)
*Maxillae*-*Pellet*: *G. clavigera*	0.95	1	28.87	<0.001
*Maxillae*-*Pellet*: *O. minus*	0.52	1	0.04	0.833

## Data Availability

The datasets generated during and/or analyzed during the current study were submitted to TreeBase. Sequence data that support the findings of this study have been deposited in GenBank. All data generated or analyzed during this study are included in this published article. All specimens described in this study were deposited in institutional herbaria.
